# Holter-ECG findings after acute ischemic stroke and TIA: A systematic analysis of the MonDAFIS randomized trial

**DOI:** 10.1038/s41598-026-54603-z

**Published:** 2026-06-01

**Authors:** Niklas Thießen, Manuel C. Olma, Serdar Tütüncü, Muhammad Jawad-Ul-Qamar, Claudia Kunze, Götz Thomalla, Darius G. Nabavi, Joachim Röther, Ulrich Laufs, Roland Veltkamp, Peter U. Heuschmann, Paulus Kirchhof, Matthias Endres, Karl Georg Haeusler, Renate B. Schnabel

**Affiliations:** 1 Department of Cardiology, University Heart and Vascular Center Hamburg, University Medical Center Hamburg- Eppendorf (UKE), Hamburg, Germany; 2 Center for Stroke Research Berlin, Charité - Universitätsmedizin Berlin, Berlin, Germany; 3 Department of Neurology, Alexianer St. Josefs-Krankenhaus Potsdam, Potsdam, Germany; 4 Medizinische Hochschule Brandenburg Theodor Fontane, Neuruppin, Germany; 5 Department of Cardiology, Castle Hill Hospital, Hull University Teaching Hospitals, NHS trust, Hull, UK; 6 Cluster of Excellence NeuroCure, Charité – University Medicine, Berlin, Germany; 7 Department of Neurology, University Medical Center Hamburg-Eppendorf, Hamburg, Germany; 8 Department of Neurology, Vivantes Klinikum Neukölln, Berlin, Germany; 9 Department of Neurology, Asklepios Klinik Altona, Hamburg, Germany; 10 Klinik und Poliklinik für Kardiologie, Universitätsklinikum Leipzig, Leipzig, Germany; 11 Department of Neurology, Alfried Krupp Krankenhaus, Essen, Germany; 12 Department of Brain Sciences, Imperial College London, London, UK; 13 Institute for Clinical Epidemiology and Biometry, University of Würzburg, Würzburg, Germany; 14 University Hospital Würzburg, Clinical Trial Center Würzburg, Würzburg, Germany; 15 Institute of Cardiovascular Sciences, College of Medical and Dental Sciences, Medical School, University of Birmingham, Birmingham, UK; 16 Department of Cardiology, UHB and SWBH NHS Trusts, Birmingham, UK; 17 German Center for Cardiovascular Research (DZHK), Partner Site North, Hamburg, Germany; 18 Klinik und Hochschulambulanz für Neurologie mit Abteilung für Experimentelle Neurologie, Charité- Universitätsmedizin Berlin, Berlin, Germany; 19 German Center for Neurodegenerative Diseases (DZNE), Partner Site Berlin, Berlin, Germany; 20 German Center for Cardiovascular Diseases (DZHK), Partner Site Berlin, Berlin, Germany; 21 partner site Berlin, German Center for Mental Health (DZPG), Berlin, Germany; 22 Department of Neurology, Universitätsklinikum Ulm, Ulm, Germany; 23 Department of Cardiology, University Heart & Vascular Center Hamburg, University Medical Center Hamburg-Eppendorf, Martinistrasse 52, 20246 Hamburg, Germany

**Keywords:** Atrial fibrillation, Stroke, ECG monitoring, Cardiology, Diseases, Medical research, Neurology

## Abstract

Holter-ECG monitoring is a critical component of post-stroke diagnostics, guiding cardiac work-up and secondary stroke prevention. Abnormal ECG findings beyond atrial fibrillation (AF) in stroke patients remain understudied. The prospective multicenter MonDAFIS trial randomized patients with acute ischemic stroke or transient ischemic attack (TIA) without known AF to Holter-ECG recording up to 7 days or usual care. Holter-ECG findings from the first 72 h of the intervention arm were analyzed to provide a reference guide in clinical practice. Furthermore, 24-hour and 72-hour Holter-ECG monitoring were compared to analyze the value of prolonged monitoring. 24-hour Holter-ECGs from 1,665 patients (median age 67; 40.4% women) identified supraventricular tachycardia (SVT) in 4.1% and newly-diagnosed AF in 2.2% of patients. Premature ventricular complexes were common (85.8%), ventricular couplets (28.0%) or bigeminy (14.0%) less common. Non-sustained ventricular tachycardia (nsVT) was detected in 1.7% of patients. Extended 72-hour-monitoring in 1,283 patients led to higher detection rates across all abnormalities, doubling nsVT (4.4%) and SVT (8.8%) detection rates. Generally, we observed higher detection rates with older age. Detection rates of supraventricular arrhythmias were higher in women, whereas men exhibited higher rates of ventricular abnormalities. Post-stroke ECG monitoring detects various arrhythmias beyond AF in a substantial proportion of individuals. Longer monitoring and older age are associated with increased detection rates, with notable sex-specific differences.

**Supplementary Information:**

The online version contains supplementary material available at 10.1038/s41598-026-54603-z.

## Introduction

Ischemic stroke constitutes one of the leading causes of mortality and disability worldwide^1^. Cardiac pathologies comprise a common comorbidity in patients with ischemic stroke with severe cardiac complications occurring in 10–20% of patients with ischemic stroke and are associated with worse long-term outcome^2–4^..

As up to one third of ischemic strokes are attributable to atrial fibrillation (AF), requiring the initiation of oral anticoagulation (OAC) for secondary prevention of suspected cardioembolic events, ECG monitoring is an essential diagnostic procedure in post-stroke care. Current guidelines recommend prolonged monitoring, often 72 h^5^,longer duration in higher risk populations has been suggested^6^..

Since the optimal duration of post-stroke ECG monitoring is uncertain, the prospective randomized multicenter MonDAFIS trial assessed whether Holter-ECG recording up to 7 days in addition to usual care would lead to a higher first-time detection rate of AF after acute ischemic stroke or transient ischemic attack (TIA)^7^. Even though a higher rate of AF detection was observed in-hospital in the intervention group, no significant difference in OAC use after 12 months was shown between groups^8^..

In addition to AF, a variety of ECG findings are frequently detected on Holter-ECG recordings. For example, premature atrial complexes (PACs) are present in the majority of healthy adults^9–11^. An increased prevalence of PACs has been reported in patients with cardiac diseases and after stroke^12,13^, and has been related to AF^14^.Others, such as ventricular ectopy are associated with underlying cardiac disease^15^. Some arrhythmias, e.g. higher degree AV block or sustained ventricular tachycardia can present as acute life threatening event after stroke^16^. However, data on findings during longer-term ECG monitoring in post-stroke patients are limited^16–18^. Previous single-center studies of smaller sample sizes reported varying frequencies of pathological ECG findings in stroke patients^19–21^..

In clinical practice physicians are often confronted with incident ECG findings that require interpretation and clinical decision-making. Therefore, in this sub-analysis of the MonDAFIS study, we aim to present a representative overview of ECG findings in a large cohort of contemporary patients with acute ischemic stroke to serve as a reference guide, specifically examining age- and sex- differences, as well as those between stroke and TIA, and between 24-hour and 72-hour ECG recordings.

## Methods

### Study population

The study’s design and rationale have been described previously^8^. We used data from the prospective multicenter “Systematic monitoring for detection of atrial fibrillation in patients with acute ischaemic stroke” (MonDAFIS) trial (NCT02204267). In total, 3,470 patients (aged at least 18 or older but without known AF) with ischemic stroke or TIA on admission were enrolled in 38 certified stroke units in Germany. Study patients were randomized 1:1 to either usual ECG monitoring (at least 24 h of monitoring combined with baseline 12-lead ECG) or extended Holter-ECG recording (LIFECARD CF recorder, Spacelabs Healthcare, Nürnberg, Germany) for up to 7 days during the in-hospital stay starting as soon as possible during the first 24 h upon stroke unit admission. ECG data were transmitted to the Institute of Cardiovascular Sciences at the University of Birmingham, Birmingham, UK for central review by physicians (PK, MJ-U-Q) or trained physiologists after receiving specific training in Holter- ECG analysis. In case of doubt or suspected clinical consequences, a physician was consulted. Data were analyzed at the Institute of Clinical Epidemiology and Biometry, University of Würzburg, Würzburg, Germany. The primary trial outcome was the percentage of patients receiving OAC 12 months after the event. Ethics committee approval was obtained from all collaborating centers, first by the Charité Ethics Committee, Berlin, Germany (EA2_033_14). All study patients gave written informed consent and treatment of patients and further analysis was performed in accordance with relevant guidelines and regulations. For this analysis, data from the intervention cohort were utilized. All 24-hour analyses were conducted on the full dataset (*N* = 1,665), whereas 72-hour analyses were restricted to participants with ≥ 72 h of analyzable recordings (*N* = 1,283) (Supplementary Fig. 1).

### Statistical analyses

The descriptive statistics are shown as n (%), median (25th;75th percentile) or mean +/- standard deviation. In order to calculate selected prevalence estimates, exact binomial 95% confidence intervals were used. Association between group classification and categorical variables were analyzed using a chi square test with a two-sided significance level of 0.05.

## Results

A total of 1,665 patients (40.4% women) from the intervention group were included in this analysis.The average duration of ECG recording of patients in the intervention group receiving cardiology core laboratory centrally reviewed Holter-ECG was 118.5 h (25th/75th percentile 74.9,166.3)^8^. We compared arrhythmia detection rates during the initial 24 h of monitoring with those obtained from patients who underwent 72-hour recording.

Most study patients were between 50 and 75 years old (61.6%), 10.5% were younger than 50 years and 27.9% were older than 75 years. The majority of study patients had a history of arterial hypertension (77.6%) and hyperlipidemia (53.1%). Heart failure, diabetes mellitus, renal impairment and vascular disease were most prevalent in the age group > 75 years. Patients in the age group < 50 years showed lowest comorbidity burden, but highest rate of smoking. Furthermore, TIA and low severity stroke (defined as NIHSS score 0 and modified Rankin Scale (mRS) ≤ 2 on admission) were highest in the age group < 50 years (Table 1, Supplementary Table 1).

**Table 1:** Overview of baseline characteristics of the subpopulation of the MonDAFIS trial with available 24 h ECG recording categorized by age groups and 72 h ECG recording. Provided are total number and percentage or median and 25th; 75th percentile, respectively.

Age (years)	24 h				72 h
	Total	< 50 years	50–75 years	> 75 years	Total
	*N* = 1,665	*N* = 175 (10.5%)	*N* = 1,026 (61.6%)	*N* = 464 (27.9%)	*N* = 1,283
Women, n (%)	672 (40.4)	71 (40.6)	368 (35.9)	233 (50.2)	508 (39.6)
Age, years	67 (57; 76)				67 (58; 76)
NIHSS on admission 0, n (%)	177 (10.7)	34 (19.7)	107 (10.5)	36 (7.8)	128 (10.0)
NIHSS on admission 1–4, n (%)	1118 (67.5)	109 (63.0)	686 (67.3)	323 (69.6)	848 (66.1)
NIHSS on admission > 4, n (%)	361 (21.8)	30 (17.3)	226 (22.2)	105 (22.6)	300 (23.4)
mRS on admission > 2, n (%)	610 (36.7)	38 (22.0)	367 (35.8)	205 (44.3)	494 (38.5)
TIA, n (%)	488 (29.4)	68 (39.3)	298 (29.1)	122 (26.3)	338 (26.3)
Endovascular thrombectomy, n (%)	39 (2.4)	5 (2.9)	20 (2.0)	14 (3.0)	33 (2.6)
Intravenous thrombolysis, n (%)	361 (21.7)	38 (22.0)	238 (23.2)	85 (18.3)	279 (21.7)
Heart failure, n (%)	48 (2.9)	1 (0.6)	36 (3.5)	11 (2.4)	33 (2.6)
COPD, n (%)	77 (4.7)	2 (1.2)	55 (5.4)	20 (4.4)	64 (5.0)
Arterial hypertension, n (%)	1282 (77.6)	66 (38.4)	818 (80.1)	398 (86.7)	1002 (78.1)
Diabetes mellitus, n (%)	441 (26.7)	17 (9.9)	284 (27,8)	140 (30.5)	352 (27.4)
Hyperlipidemia, n (%)	877 (53.1)	65 (37.8)	561 (54.9)	251 (54.7)	671 (52.3)
Renal impairment, n (%)	128 (7.7)	1 (0.6)	56 (5.5)	71 (15.5)	103 (8.0)
Vascular disease, n (%)	237 (14.4)	3 (1.7)	144 (14.1)	90 (19.7)	186 (14.5)
Prior cardiovascular event, n (%)	405 (24.5)	15 (8.7)	247 (24.2)	143 (31.3)	314 (24.5)
Smoking, n (%)	826 (49.9)	110 (63.6)	586 (57.4)	130 (28.2)	617 (48.1)
BMI > 30 kg/m², n (%)	412 (25.0)	54 (31.2)	270 (26.6)	88 (19.2)	323 (25.2)
Duration of recording (hours), n (%)	121.7 (74.9; 166.2)				
Artefact percentage, n (25th/75th percentile)	2.05 (0.7; 4.9)				
Minimum heart rate, n (25th/75th percentile)	51 (47; 56)				
Maximum heart rate, n (25th/75th percentile)	112.5 (100;125)				

Age composition differed significantly between men and women with a higher proportion of patients > 75 years being female (24.7%) in comparison to male (23.3%). Generally, female patients were significantly less likely to have vascular disease (8.3%, *p* < 0.0001), prior cardiovascular events (19.9%, *p* = 0.0002), diabetes mellitus (19.5%, *p* < 0.0001) or a history of smoking (19.9%, *p* = 0.0002) (Supplementary Tables 1–2, Supplementary Fig. 3–4).

In comparison to stroke patients, those suffering from a TIA had a lower comorbidity burden (Supplementary Table 3, Supplementary Fig. 5).

During the first 24 h of study-related ECG monitoring, frequently observed findings included premature atrial complexes (PACs, 98.1%), with SV couplets (68.2%) and SV ectopic runs (55.0%). SVT was present in 4.4% of patients and the AF detection rate after 24 h was 2.2%. (Table 2).

**Table 2:** Overview of supraventricular ECG abnormalities within the first 24 and 72 h of monitoring, respectively. Provided are total number (% with 95% confidence interval) or median (25th; 75th percentile), respectively.

ECG findings	24 h *N* = 1,665	72 h *N* = 1,283
Premature atrial complexes, n (%; 95% CI)	1,633 (98.1; 95% CI: 97.3–98.7)	1,275 (99.4; (95% CI: 98.8–99.7)
No. of PAC	50 (14; 256)	
Supraventricular ectopic couplets, n (%; 95% CI)	1,135 (68.2; 95% CI: 65.9–70.4)	1,066 (83.1; (95% CI: 80.9–85.1)
Supraventricular ectopic run, n (%; 95% CI)	915 (55.0; 95% CI: 52.5–57.4)	939 (73.2; (95% CI: 70.7–75.6)
No. of SVE runs (among SVE run positive)	3 (1; 8)	5 (2; 15)
Average duration (sec.)	1.57 (1.2; 2.1)	1.59 (1.3; 2.1)
Longest SVE run (sec.)	2.29 (1.4; 4.0)	2.81 (1.7; 5.0)
Sum of all SVE runs (sec.)	5.28 (2.1; 15.3)	9.27 (3.4; 30.0)
Supraventricular tachycardia (SVT) n (%; 95% CI)	68 (4.1; 95% CI: 3.2–5.1)	113 (8.8; (95% CI: 7.3–10.5)
No. of SVTs (among SVT positive)	1 (1; 2)	1 (1; 2)
Average SVT duration (sec.)	3.4 (2.0; 11.6)	3.4 (2.0; 9.3)
Longest SVT (sec.)	3.9 (2.0; 13.1)	4.0 (2.2; 11.3)
Sum of all SVTs (sec.)	5.2 (2.2; 17.0)	5.4 (2.7; 15.4)
Atrial fibrillation (AF), n (%; 95% CI)	36 (2.2; 95% CI: 1.5–3.0)	39 (3.0; 95% CI: 2.2–4.1)
No. of AF episodes (among AF positive)	3 (2; 7)	4 (1; 12)
Average duration of AF episodes (min.)	36 (2.2; 338)	74 (3.1; 413)
Longest AF episode (min.)	148 (6.1; 757)	197 (10; 703)
Sum of all AF episodes (min.)	364 (9.2; 1,015)	382 (32; 1,148)

Premature ventricular complexes (PVCs) were detected in 85.8% of patients, ventricular couplets in 28.0% and triplets in 9.1%. Nonsustained VT was detected in 1.7% of patients, however no sustained VT was detected (Table 3).

**Table 3:** Overview of ventricular ECG abnormalities within the first 24 and 72 h of monitoring. Provided are total number (% with 95% confidence interval) or median (25th; 75th percentile), respectively.

ECG findings	24 h *N* = 1,665	72 h *N* = 1,283
Premature ventricular complex (PVC); n (%; 95% CI)	1,428 (85.8; 95% CI: 84.0–87.4)	1,211 (94.4; 95% CI: 93.0–95.6)
No. of PVC	17 (2; 150)	
Ventricular bigeminy	233 (14.0; 95% CI: 12.4–15.8)	296 (23.1; 95% CI: 20.8–25.5)
No. of bigeminy (among bigeminy positive)	5 (1; 31)	5 (2; 35)
Average bigeminy duration (sec.)	3.9 (3.1; 5.00)	3.7 (3.1; 4.7)
Longest bigeminy (sec.)	5.2 (3.5; 10.9)	5.3 (3.4; 10.0)
Sum of all bigeminy episodes (sec.)	17 (4; 138)	17 (6; 138)
Ventricular trigeminy	241 (14.5; 95% CI: 12.8–16.3)	280 (21.8; 95% CI: 19.6–24.2)
No. of trigeminy (among trigeminy positive)	7 (2; 42)	8 (2; 46)
Average trigeminy duration (sec.)	6.3 (4.9; 8.5)	5.9 (4.8; 7.8)
Longest bigeminy (sec.)	8.9 (5.5; 27.3)	9.4 (5.4; 23.0)
Sum of all trigeminy episodes (sec.)	45 (9; 330)	48 (9; 351)
R-on-T phenomenon	84 (5.0; 95% CI: 4.0–6.2)	120 (9.4; 95% CI: 7.8–11.1)
Ventricular run/accelerated idioventricular rhythm (AIVR)	97 (5.8; 95% CI: 4.7–7.1)	166 (12.9; 95% CI: 11.1–14.9)
Ventricular ectopic couplet	466 (28.0; 25.8–30.2)	534 (41.6; 95% CI: 38.9–44.4)
Ventricular ectopic triplet	152 (9.1; 95% CI: 7.8–10.6)	218 (17.0; 95% CI: 15.0–19.2)
Ventricular tachycardia (VT)	28 (1.7; 95% CI: 1.1–2.4)	56 (4.4; 95% CI: 3.3–5.6)
No. of VTs (among VT positive)	1 (1; 1)	1 (1; 1)
Average VT duration (sec.)	2.1 (1.4; 5.4)	2.00 (1.4; 4.6)
Longest VT (sec.)	2.1 (1.4; 6.0)	2.5 (1.4; 5.9)
Sum of all VT episodes (sec.)	2 (1; 6)	3 (1; 6)

Bradycardia was observed in 20.2% of patients. 2.0% of patients showed pauses with a median duration of 2.9 (25th/75th percentile 2.55; 3.61) seconds. Episodes of tachycardia were detected in 14.7% of patients (Table 4).

**Table 4:** Overview of further ECG abnormalities within the first 24 and 72 h of monitoring, respectively. Provided are total number (% with 95% confidence interval) or median (25th; 75th percentile), respectively.

ECG findings	24 h *N* = 1,665	72 h *N* = 1,283
Tachycardia	245 (14.7; 95% CI: 13.0–16.5)	324 (25.3; 95% CI: 22.9–27.7)
No. of tachycardias (among tachycardia positive)	8 (2; 33)	12 (4; 41)
Average tachycardia duration (sec.)	6.1 (2.7; 11.0)	5.6 (2.8; 11.1)
Longest tachycardia duration (sec.)	19.8 (5.0; 58.7)	25.6 (5.9; 62.1)
Sum of all tachycardias (sec.)	60 (12; 256)	97 (22; 347)
Bradycardia	336 (20.2; 95% CI: 18.3–22.2)	383 (29.9; 95% CI: 27.4–32.4)
No. of bradycardias (among bradycardia positive)	23 (3; 176)	29 (3; 245)
Average bradycardia duration (sec.)	6.9 (5.4; 10.1)	6.6 (5.4; 9.3)
Longest bradycardia (sec.)	16.5 (8.2; 40.0)	16.9 (7.6; 43.8)
Sum of all bradycardias (sec.)	161 (23; 1671)	194 (22; 2110)
Pause	33 (2.0; 95% CI: 1.4–2.8)	41 (3.2; 95% CI: 2.3–4.3)
No. of pauses (among pause positive)	2 (1; 12)	3 (1; 8)
Average pause duration (sec.)	2.9 (2.6; 3.6)	2.9 (2.6; 3.6)
Longest pause (sec.)	3.5 (2.6; 4.3)	3.5 (2.6; 4.7)
Sum of all pauses (sec.)	8 (4; 54)	10 (5; 29)
Supraventricular escape (SVEsc)	430 (25.8; 95% CI: 23.7–28.0)	448 (34.9; 95% CI: 32.3–37.6)
Ventricular escape (VEsc)	118 (7.1; 95% CI: 5.9–8.4)	180 (14.0; 95% CI: 12.2–16.1)

**Fig. 1:**
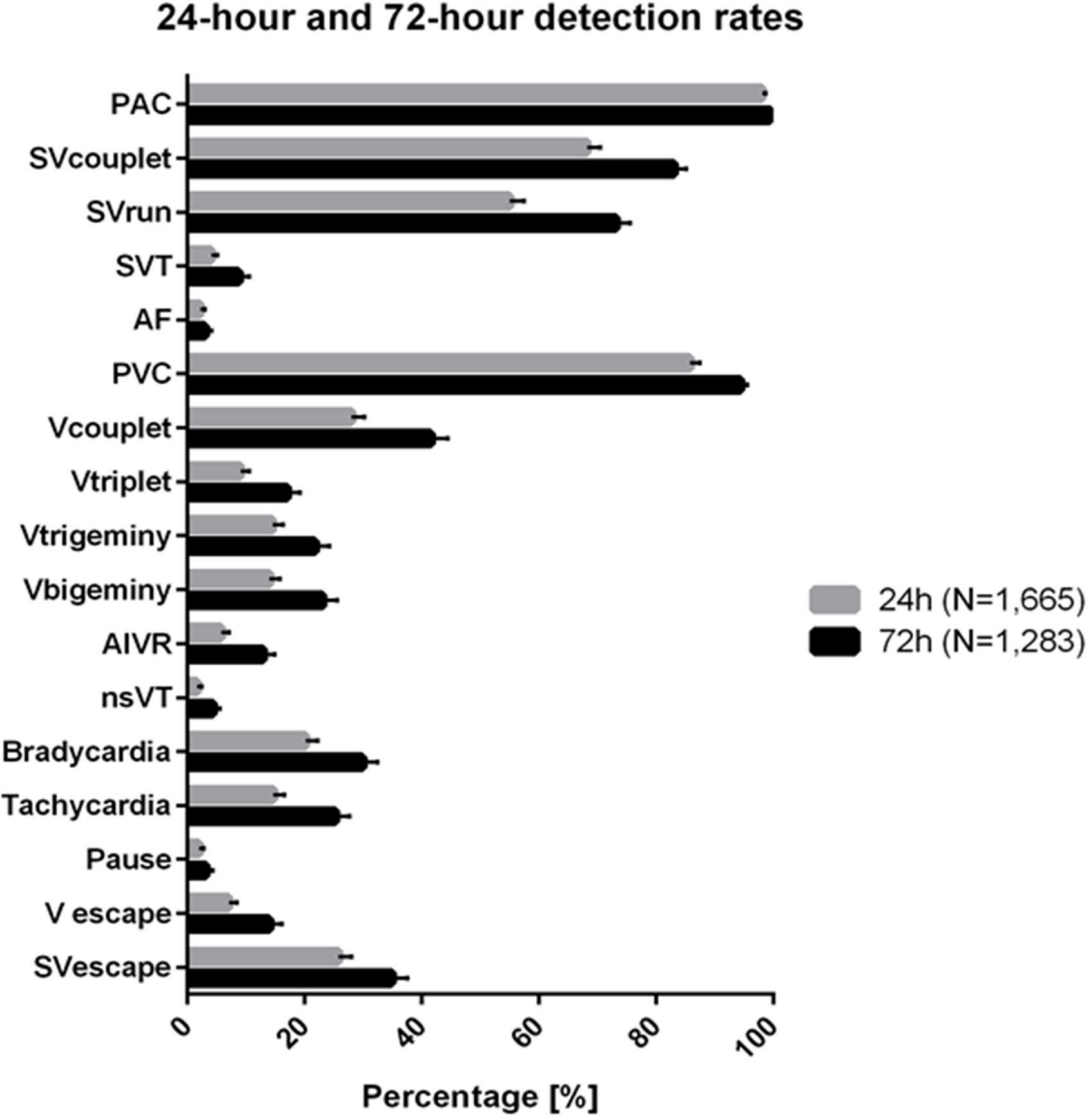
Schematic overview of detection rates of arrhythmias as percentage of the respective patient population in both the 24-hour (*N* = 1,665) and 72-hour (*N* = 1,283) group. 95% confidence intervals are provided as error bars.

Across all ECG findings of varying clinical significance, we observed an increased detection rate after 72 h in comparison to the first 24 h of monitoring. The detection rate of atrial fibrillation rose from 2.2% to 3.0%, SVT detection from 4.1% to 8.8% and nsVT detection from 1.7% to 4.4% (Fig. 1; Supplementary Fig. 2**)**.

With respect to the different age groups (< 50 years, 50–75 years, > 75 years), we observed highest detection rates across almost all ECG findings for the older age groups with no AF detection in those patients under 50 years old. Outliers in this regard were detection rates of tachycardia and bradycardia with highest detection rates for those patients < 50 years (Supplementary Table 4).

Detection rates of supraventricular arrhythmias (e.g. SVT, AF) and tachycardia were higher in women, whereas men exhibited higher detection rate of ventricular abnormalities (nsVT, bigeminy, couplets) as well as bradycardia and pauses. (Supplementary Table 5).

Differentiation between TIA and stroke cases yielded higher rates of bradycardia (26.4%; 17.6% in stroke), as well as supraventricular (28.7%; 24.6% in stroke) and ventricular escape rhythms (8.2%; 6.6% in stroke) in TIA patients (Supplementary Table 6).

## Discussion

In an all-comer study of patients with acute ischemic stroke of mild to moderate clinical severity or TIA, continuous ECG monitoring of up to 72 h yielded a rather high proportion of rhythm abnormalities of potential clinical relevance. Given that ischemic stroke and cardiac diseases are driven by similar cardiovascular risk factors, co-morbidity and coincidence of cardiac conditions in post-stroke patients is common^2^. Furthermore, elevated risk for cardiovascular death after stroke survival has been observed^3^. Currently, data on the prevalence of abnormal ECG findings in post-stroke patients is scarce and lacks a standard, as previous studies are of small sample sizes, limited in generalizability and prone to selection bias^17–21^. Our work can provide an overview of ECG findings to serve as a reference for daily clinical practice.

In the general population PVCs are a common finding with a reported prevalence of 40–75% in 24–48 h of Holter-ECG monitoring^22^. We observed an even higher prevalence of PVC (85.8%) after 24 h with an increase to 94.4% with 72-hour monitoring. In contrast, VT were rarely detected and, when present, of short duration (median 2.08 s). In comparison, the contemporary Copenhagen Holter study yielded a higher detection rate of 10.6% upon Holter-ECG recording of a least 48 h in middle-aged and elderly individuals without known heart disease^23^. VT detection should prompt a thorough cardiac workup, as VT in the presence of structural heart disease, e.g. post myocardial infarction, is associated with sudden cardiac death^24,25^. However, in the absence of heart disease even presence of non-sustained VT can be a relatively benign finding^26^. In general, the underlying conditions of VT determine the prognostic impact and require careful cardiovascular work-up^27,28^. Among all patients who underwent study protocol Holter-ECG monitoring (*N*= 1,693), second-degree atrioventricular (AV) block was detected in only 11 patients (0.6%), and no cases of complete AV block were observed^29^..

Overall, 72-hour ECG monitoring compared to 24 h only, led to an increased detection rate across all arrhythmias under investigation. Whether the additionally gained information is of clinical relevance (Supplementary Table 7) and related to outcomes, needs to be examined. Sex specific differences were observed in both baseline characteristics and ECG findings. Overall, higher rates of supraventricular arrhythmias were detected in female patients, whereas male patients exhibited higher rates of ventricular arrhythmias (Supplementary Table 5). The higher detection rate of supraventricular arrhythmias in women is consistent with previous studies demonstrating a greater proportion of cardioembolic strokes among female patients^30^. In contrast, men were more likely to have underlying vascular disease, corresponding to a higher proportion of atherothrombotic stroke etiology in previous studies^31^. Furthermore, major differences in detection rates were observed when comparing different age groups, with older patients exhibiting more ECG abnormalities.

### Limitations

While the current study offers a reference guide of the observed frequency of ECG abnormalities in a contemporary cohort of patients with acute ischemic stroke of mild to moderate clinical severity or TIA, it does not answer the question of the clinical relevance of said abnormalities as a we did not assess outcomes. To which extent these findings reflect the physiological aging process as opposed to being driven by comorbidities and to what extent they should prompt further diagnostic workup warrants further research. Generally, the open-label design of the MonDAFIS trial is prone to selection bias, thus generalizability of our findings to other post stroke patient populations might be limited. External validity is further constrained by heterogeneity in monitoring duration and differences in study populations among Holter-ECG trials.

## Conclusions

Pathological ECG findings are frequently observed during routine monitoring after acute ischemic stroke or TIA. Our findings provide a reference guide of ECG characteristics in a contemporary, not severely disabled stroke cohort. Notably, while many ECG changes may be benign, they can also reflect serious underlying cardiac disease and therefore warrant careful diagnostic evaluation.

## Electronic Supplementary Material

Below is the link to the electronic supplementary material.

Supplementary Material 1

## Data Availability

Deidentified participant data with corresponding data dictionary of the data underlying the current manuscript will be made available on reasonable request to the corresponding author after publication of all secondary endpoints. Additional documents such as the study protocol and statistical analysis plan will also be available on request. The data will be shared to external researchers for scientific non-commercial purposes with investigator support after approval of the proposal by the MonDAFIS study steering board, including a signed data access agreement.

## Abbreviations

AF: Atrial Fibrillation
OAC: Oral Anticoagulant

## References

[1] Campbell, B. C. V. et al. Ischaemic stroke. *Nat. Rev. Dis. Primers* **5**, 70. 10.1038/s41572-019-0118-8 (2019). 31601801 10.1038/s41572-019-0118-8

[2] Scheitz, J. F. et al. Stroke–Heart Syndrome: Recent advances and challenges. *J. Am. Heart Assoc.* **11**, e026528. 10.1161/JAHA.122.026528 (2022). 36056731 10.1161/JAHA.122.026528 PMC9496419

[3] Hankey, G. J. et al. Five-year survival after first-ever stroke and related prognostic factors in the Perth Community Stroke Study. *Stroke* **31**, 2080–2086. 10.1161/01.str.31.9.2080 (2000). 10978033 10.1161/01.str.31.9.2080

[4] Rutten-Jacobs, L. C. et al. Long-term mortality after stroke among adults aged 18 to 50 years. *JAMA* **309**, 1136–1144. 10.1001/jama.2013.842 (2013). 23512060 10.1001/jama.2013.842

[5] Van Gelder, I. C. et al. ESC Guidelines for the management of atrial fibrillation developed in collaboration with the European Association for Cardio-Thoracic Surgery (EACTS): Developed by the task force for the management of atrial fibrillation of the European Society of Cardiology (ESC), with the special contribution of the European Heart Rhythm Association (EHRA) of the ESC. Endorsed by the European Stroke Organisation (ESO). *European Heart Journal*, ehae176, (2024). 10.1093/eurheartj/ehae176 (2024). 10.1093/eurheartj/ehae176 39210723

[6] Schnabel, R. B. et al. Searching for Atrial Fibrillation Poststroke: A White Paper of the AF-SCREEN International Collaboration. *Circulation* **140**, 1834–1850. 10.1161/circulationaha.119.040267 (2019). 31765261 10.1161/CIRCULATIONAHA.119.040267

[7] Haeusler, K. G. et al. Impact of standardized MONitoring for Detection of Atrial Fibrillation in Ischemic Stroke (MonDAFIS): Rationale and design of a prospective randomized multicenter study. *Am. Heart J.* **172**, 19–25. 10.1016/j.ahj.2015.10.010 (2016). 26856211 10.1016/j.ahj.2015.10.010

[8] Haeusler, K. G. et al. Systematic monitoring for detection of atrial fibrillation in patients with acute ischaemic stroke (MonDAFIS): A randomised, open-label, multicentre study. *Lancet. Neurol.* **20**, 426–436. 10.1016/S1474-4422(21)00067-3 (2021). 34022169 10.1016/S1474-4422(21)00067-3

[9] Folarin, V. A., Fitzsimmons, P. J. & Kruyer, W. B. Holter monitor findings in asymptomatic male military aviators without structural heart disease. *Aviat. Space Environ. Med.* **72**, 836–838 (2001). 11565820

[10] Camm, A. J., Evans, K. E., Ward, D. E. & Martin, A. The rhythm of the heart in active elderly subjects. *Am. Heart J.* **99**, 598–603. 10.1016/0002-8703(80)90733-4 (1980). 7369099 10.1016/0002-8703(80)90733-4

[11] Fleg, J. L. & Kennedy, H. L. Cardiac arrhythmias in a healthy elderly population: Detection by 24-hour ambulatory electrocardiography. *Chest* **81**, 302–307. 10.1378/chest.81.3.302 (1982). 7056104 10.1378/chest.81.3.302

[12] Chong, B. H. et al. Frequent premature atrial complexes predict new occurrence of atrial fibrillation and adverse cardiovascular events. *Europace: Eur. pacing Arrhythm. cardiac Electrophysiol. : J. working groups cardiac pacing Arrhythm. cardiac Cell. Electrophysiol. Eur. Soc. Cardiol.* **14**, 942–947. 10.1093/europace/eur389 (2012). 10.1093/europace/eur389 22183750

[13] Haeusler, K. G. et al. Excessive supraventricular ectopic activity in patients with acute ischemic stroke is associated with atrial fibrillation detection within 24 months after stroke: A predefined analysis of the MonDAFIS Study. *J. Am. Heart Assoc.* **14**, e034512. 10.1161/JAHA.123.034512 (2025). 39791425 10.1161/JAHA.123.034512 PMC12054430

[14] Chousou, P. A., Chattopadhyay, R., Tsampasian, V., Vassiliou, V. S. & Pugh, P. J. Electrocardiographic predictors of atrial fibrillation. *Medical sciences (Basel, Switzerland)*10.3390/medsci11020030 (2023). 37092499 10.3390/medsci11020030 PMC10123668

[15] Soliman, E. Z., Elsalam, M. A. & Li, Y. The relationship between high resting heart rate and ventricular arrhythmogenesis in patients referred to ambulatory 24 h electrocardiographic recording. *EP Europace*. **12**, 261–265. 10.1093/europace/eup344 (2010). 19887457 10.1093/europace/eup344

[16] Kallmünzer, B. et al. Serious cardiac arrhythmias after stroke: incidence, time course, and predictors–a systematic, prospective analysis. *Stroke* **43**, 2892–2897. 10.1161/strokeaha.112.664318 (2012). 22961962 10.1161/STROKEAHA.112.664318

[17] Haeusler, K. G., Tütüncü, S. & Schnabel, R. B. Detection of atrial fibrillation in cryptogenic stroke. *Curr. Neurol. Neurosci. Rep.* **18**, 66. 10.1007/s11910-018-0871-1 (2018). 30090997 10.1007/s11910-018-0871-1

[18] Sposato, L. A., Lip, G. Y. H. & Haeusler, K. G. Atrial fibrillation first detected after stroke: Is timing and detection intensity relevant for stroke risk?. *Eur. Heart J.* **45**, 396–398. 10.1093/eurheartj/ehad744 (2023). 10.1093/eurheartj/ehad744 38014646

[19] Turakhia, M. P. et al. Feasibility of extended ambulatory electrocardiogram monitoring to identify silent atrial fibrillation in high-risk patients: The Screening Study for Undiagnosed Atrial Fibrillation (STUDY-AF). *Clin. Cardiol.* **38**, 285–292. 10.1002/clc.22387 (2015). 25873476 10.1002/clc.22387 PMC4654330

[20] Carrarini, C. et al. ECG monitoring of post-stroke occurring arrhythmias: An observational study using 7-day Holter ECG. *Sci. Rep.* **12**, 228. 10.1038/s41598-021-04285-6 (2022). 34997171 10.1038/s41598-021-04285-6 PMC8741921

[21] Chen, W. C. et al. Comparison of continuous 24-hour and 14-day ECG monitoring for the detection of cardiac arrhythmias in patients with ischemic stroke or syncope. *Clin. Cardiol.* **47**, e24247. 10.1002/clc.24247 (2024). 38450794 10.1002/clc.24247 PMC10918718

[22] Ahn, M. S. Current Concepts of Premature Ventricular Contractions. *J. lifestyle Med.* **3**, 26–33 (2013). 26064834 PMC4390755

[23] Sadjadieh, G. & Sajadieh, A. Prognosis After Finding Incidental Ventricular Tachycardia on Ambulatory Electrocardiogram-recording. *Am. J. Cardiol.* **150**, 60–64. 10.1016/j.amjcard.2021.03.049 (2021). 34001341 10.1016/j.amjcard.2021.03.049

[24] Scirica, B. M. et al. Relationship between nonsustained ventricular tachycardia after non–ST-elevation acute coronary syndrome and sudden cardiac death. *Circulation* **122**, 455–462. 10.1161/CIRCULATIONAHA.110.937136 (2010). 20644019 10.1161/CIRCULATIONAHA.110.937136

[25] Samuel, M., Elsokkari, I. & Sapp, J. L. Ventricular tachycardia burden and mortality: Association or causality?. *Can. J. Cardiol.* **38**, 454–464. 10.1016/j.cjca.2022.01.016 (2022). 35074416 10.1016/j.cjca.2022.01.016

[26] Marine, J. E. et al. Prevalence and prognostic significance of exercise-induced nonsustained ventricular tachycardia in asymptomatic volunteers: BLSA (Baltimore Longitudinal Study of Aging). *J. Am. Coll. Cardiol.* **62**, 595–600. 10.1016/j.jacc.2013.05.026 (2013). 23747767 10.1016/j.jacc.2013.05.026 PMC3800197

[27] Frolkis, J. P., Pothier, C. E., Blackstone, E. H. & Lauer, M. S. Frequent ventricular ectopy after exercise as a predictor of death. *N. Engl. J. Med.* **348**, 781–790. 10.1056/NEJMoa022353 (2003). 12606732 10.1056/NEJMoa022353

[28] Annane, D. et al. Incidence and prognosis of sustained arrhythmias in critically ill patients. *Am. J. Respir. Crit. Care Med.* **178**, 20–25. 10.1164/rccm.200701-031OC (2008). 18388358 10.1164/rccm.200701-031OC

[29] Olma, M. C. et al. In-hospital ECG findings, changes in medical management, and cardiovascular outcomes in patients with acute stroke or transient ischemic attack. *J. Am. Heart Assoc.* **12**, e027149. 10.1161/JAHA.122.027149 (2023). 36628982 10.1161/JAHA.122.027149 PMC9939074

[30] Roquer, J., Campello, A. R. & Gomis, M. Sex differences in first-ever acute stroke. *Stroke* **34**, 1581–1585. 10.1161/01.str.0000078562.82918.f6 (2003). 12805490 10.1161/01.STR.0000078562.82918.F6

[31] Förster, A. et al. Gender differences in acute ischemic stroke: Etiology, stroke patterns and response to thrombolysis. *Stroke* **40**, 2428–2432. 10.1161/strokeaha.109.548750 (2009). 19461021 10.1161/STROKEAHA.109.548750

