# Supplementary material for: Holter-ECG findings after acute ischemic stroke and TIA: A systematic analysis of the MonDAFIS randomized trial
Source: Sci Rep. 2026 Jun 1;16:16812. doi: 10.1038/s41598-026-54603-z (PMC13226722; doi:10.1038/s41598-026-54603-z)
Supplement: Supplementary file 1 — Supplementary Material 1 [file 41598_2026_54603_MOESM1_ESM.docx]

Supplementary Material

**Supplementary Figure 1.** Flow chart of MonDAFIS trial and this subanalysis’ patient population.

***
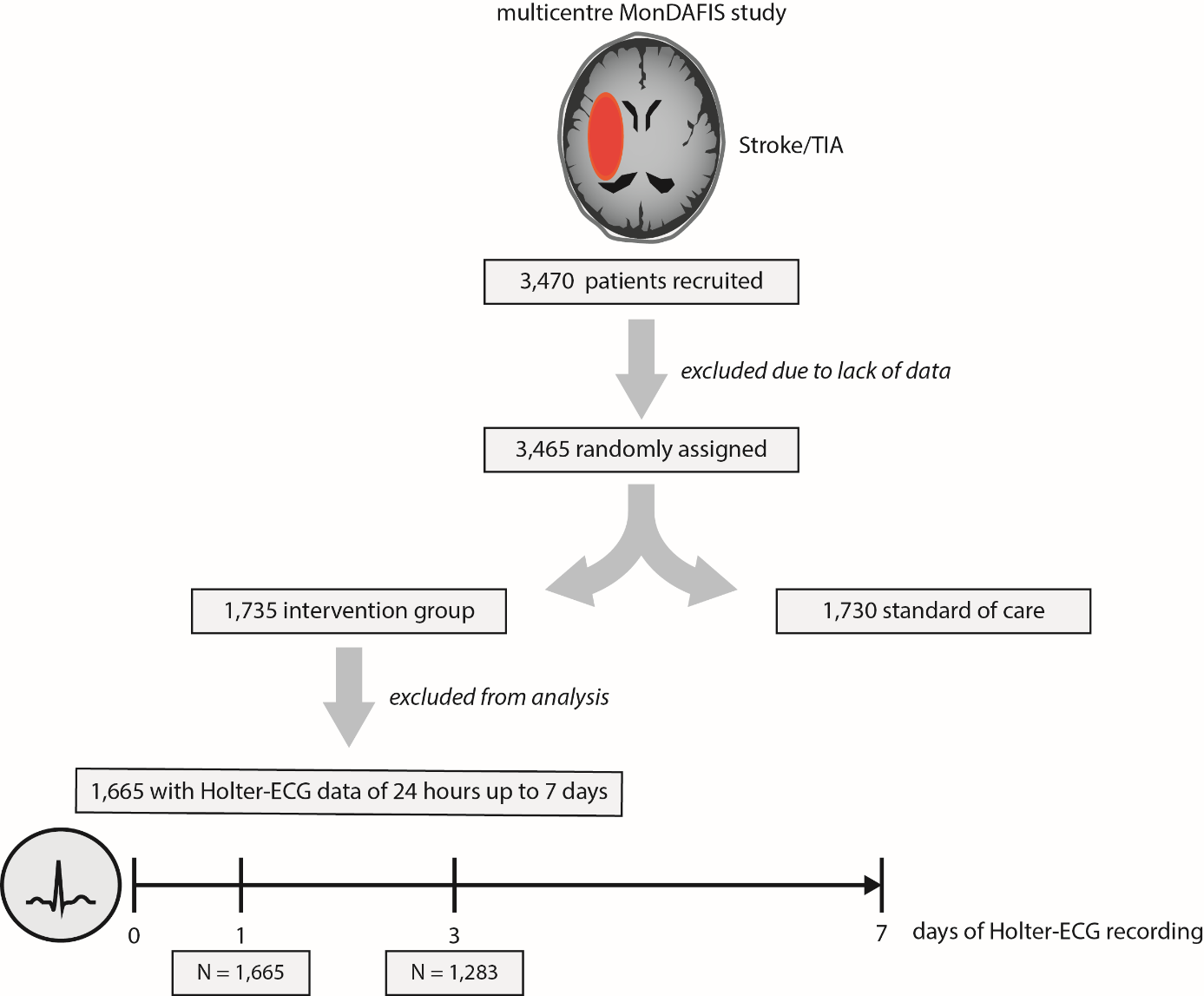
***

**Supplementary Figure 2.** Overview of incremental relative detection rate of 72-hour monitoring in comparison to 24 hours for selected ECG findings. Abbreviations: AF – atrial fibrillation, VT – ventricular tachycardia, SVT – supraventricular tachycardia, PVC – premature ventricular complex, PAC – premature atrial complex.

**Supplementary Figure 3.** Overview of age-specific detection rates [%] of selected Holter ECG findings upon 24 hours of recording. Statistical significance as calculated by chi square test is displayed with ns ≙ p>0.05; * ≙ p≤0.05; ** ≙ p≤0.01; *** ≙ p≤0.001; **** ≙ p≤0.0001. Abbreviations: PAC – Premature atrial complexes, SVcouplet – Supraventricular ectopic couplets, SVrun – Supraventricular ectopic run, SVT – supraventricular tachycardia, AF – atrial fibrillation, PVC – Premature ventricular complex, Vcouplet – Ventricular ectopic couplet, Vbigeminy – Ventricular bigeminy, nsVT – non-sustained ventricular tachycardia**

**

**Supplementary Figure 4.** Overview of sex-specific detection rates [%] of selected Holter ECG findings upon 24 hours of recording. Statistical significance as calculated by chi square test is displayed with ns ≙ p>0.05; * ≙ p≤0.05; ** ≙ p≤0.01 ; *** ≙ p≤0.001; **** ≙ p≤0.0001. Abbreviations: PAC – Premature atrial complexes, SVcouplet – Supraventricular ectopic couplets, SVrun – Supraventricular ectopic run, SVT – supraventricular tachycardia, AF – atrial fibrillation, PVC – Premature ventricular complex, Vcouplet – Ventricular ectopic couplet, Vbigeminy – Ventricular bigeminy, nsVT – non-sustained ventricular tachycardia**

**

**Supplementary Figure 5.** Overview of TIA- and stroke-specific detection rates [%] of selected Holter ECG findings upon 24 hours of recording. Statistical significance as calculated by chi square test is displayed with ns ≙ p>0.05; * ≙ p≤0.05; ** ≙ p≤0.01 ; *** ≙ p≤0.001; **** ≙ p≤0.0001. Abbreviations: PAC – Premature atrial complexes, SVcouplet – Supraventricular ectopic couplets, SVrun – Supraventricular ectopic run, SVT – supraventricular tachycardia, AF – atrial fibrillation, PVC – Premature ventricular complex, Vcouplet – Ventricular ectopic couplet, Vbigeminy – Ventricular bigeminy, nsVT – non-sustained ventricular tachycardia**

**

**Supplementary Table 1.** Overview of baseline characteristics of the subpopulation of the MonDAFIS trial with available 24 hour ECG recording categorized by age groups. Provided are total number and percentage or median and 25th; 75th percentile and p-values as per chi square test.

|  | **24 hours** | | | |  |
| --- | --- | --- | --- | --- | --- |
| **Age (years)** | **Total** | **<50 years** | **50-75 years** | **>75 years** | **p-value** |
|  | **N = 1,665** | **N = 175**  **(10.5%)** | **N = 1,026**  **(61.6%)** | **N = 464**  **(27.9%)** |  |
| Women, n (%) | 672 (40.4) | 71 (40.6) | 368 (35.9) | 233 (50.2) | < 0.0001 |
| NIHSS on admission 0, n (%) | 177 (10.7) | 34 (19.7) | 107 (10.5) | 36 (7.8) | 0.0007 |
| NIHSS on admission 1-4, n (%) | 1118 (67.5) | 109 (63.0) | 686 (67.3) | 323 (69.6) |  |
| NIHSS on admission >4, n (%) | 361 (21.8) | 30 (17.3) | 226 (22.2) | 105 (22.6) |  |
| mRS on admission >2, n (%) | 610 (36.7) | 38 (22.0) | 367 (35.8) | 205 (44.3) | < 0.0001 |
| TIA, n (%) | 488 (29.4) | 68 (39.3) | 298 (29.1) | 122 (26.3) | 0.0076 |
| Endovascular thrombectomy, n (%) | 39 (2.4) | 5 (2.9) | 20 (2.0) | 14 (3.0) | 0.4026 |
| Intravenous thrombolysis, n (%) | 361 (21.7) | 38 (22.0) | 238 (23.2) | 85 (18.3) | 0.3330 |
| Heart failure, n (%) | 48 (2.9) | 1 (0.6) | 36 (3.5) | 11 (2.4) | 0.0739 |
| COPD, n (%) | 77 (4.7) | 2 (1.2) | 55 (5.4) | 20 (4.4) | 0.0456 |
| Arterial hypertension, n (%) | 1282 (77.6) | 66 (38.4) | 818 (80.1) | 398 (86.7) | < 0.0001 |
| Diabetes mellitus, n (%) | 441 (26.7) | 17 (9.9) | 284 (27,8) | 140 (30.5) | < 0.0001 |
| Hyperlipidemia, n (%) | 877 (53.1) | 65 (37.8) | 561 (54.9) | 251 (54.7) | < 0.0001 |
| Renal impairment, n (%) | 128 (7.7) | 1 (0.6) | 56 (5.5) | 71 (15.5) | < 0.0001 |
| Vascular disease, n (%) | 237 (14.4) | 3 (1.7) | 144 (14.1) | 90 (19.7) | < 0.0001 |
| Prior cardiovascular event, n (%) | 405 (24.5) | 15 (8.7) | 247 (24.2) | 143 (31.3) | < 0.0001 |
| Smoking, n (%) | 826 (49.9) | 110 (63.6) | 586 (57.4) | 130 (28.2) | < 0.0001 |
| BMI >30kg/m², n (%) | 412 (25.0) | 54 (31.2) | 270 (26.6) | 88 (19.2) | 0.0014 |

**Supplementary Table 2.** Overview of baseline characteristics of the subpopulation of the MonDAFIS trial used for 24 hour ECG analysis categorized by sex. Provided are total number and percentage or median (25^th^; 75^th^ percentile) and p-value as calculated by chi square test.

|  | **Total** | **Men** | **Women** | **p-value** |
| --- | --- | --- | --- | --- |
|  | **N = 1,665** | **N = 993 (59.6%)** | **N = 672 (40.4%)** |  |
| Age, years | 67 (57; 76) | 66 (56; 75) | 70 (59; 78) |  |
| Age <50 years, n (%) | 175 (10.5) | 104 (10.5) | 71 (10.6) | < 0.0001 |
| Age 50 – 75 years, n (%) | 1,026 (61.6) | 658 (66.3) | 368 (54.8) |  |
| Age >75 years, n (%) | 464 (27.9) | 231 (23.3) | 233 (34.7) |  |
| NIHSS on admission 0, n (%) | 177 (10.7) | 89 (9.0) | 88 (13.2) | 0.0234 |
| NIHSS on admission 1-4, n (%) | 1,118 (67.5) | 677 (68.5) | 441 (66.1) |  |
| NIHSS on admission >4, n (%) | 361 (21.8) | 223 (22.5) | 138 (20.7) |  |
| mRS on admission >2, n (%) | 610 (36.7) | 362 (36.5) | 248 (37.1) | 0.8518 |
| TIA, n (%) | 488 (29.4) | 274 (27.6) | 214 (31.9) | 0.0615 |
| Endovascular thrombectomy, n (%) | 39 (2.4) | 20 (2.0) | 19 (2.9) | 0.2817 |
| Intravenous thrombolysis, n (%) | 361 (21.7) | 215 (21.7) | 146 (21.8) | 0.9711 |
| Heart failure, n (%) | 48 (2.9) | 32 (3.2) | 16 (2.4) | 0.3140 |
| COPD, n (%) | 77 (4.7) | 40 (4.0) | 37 (5.6) | 0.1589 |
| Arterial hypertension, n (%) | 1,282 (77.6) | 774 (78.3) | 508 (76.5) | 0.2635 |
| Diabetes mellitus, n (%) | 441 (26.7) | 311 (31.4) | 130 (19.5) | < 0.0001 |
| Hyperlipidemia, n (%) | 877 (53.1) | 531 (53.7) | 346 (52.0) | 0.4258 |
| Renal impairment, n (%) | 128 (7.7) | 76 (7.7) | 52 (7.8) | 0.9494 |
| Vascular disease, n (%) | 237 (14.4) | 181 (18.3) | 56 (8.3) | < 0.0001 |
| Prior cardiovascular event, n (%) | 405 (24.5) | 273 (27.7) | 132 (19.9) | 0.0002 |
| Smoking, n (%) | 826 (49.9) | 582 (58.8) | 244 (36.6) | < 0.0001 |
| BMI >30kg/m², n (%) | 412 (25.0) | 254 (25.8) | 158 (23.9) | 0.3376 |

**Supplementary Table 3.** Overview of baseline characteristics of the subpopulation of the MonDAFIS trial in the 24 hour ECG analysis by stroke and TIA. Provided are total number with percentage and p-value as calculated by chi square test.

|  | **Total** | **TIA** | **Stroke** | **p-value** |
| --- | --- | --- | --- | --- |
|  | **N = 1,661** | **N = 488 (29.4%)** | **N = 1,173 (70.6%)** |  |
| Women, n (%) | 670 (40.3) | 214 (43.9) | 456 (38.9) | 0.0596 |
| Age <50, n (%) | 173 (10.4) | 68 (13.9) | 105 (9.0) | 0.0058 |
| Age 50 – 75, n (%) | 1,025 (61.7) | 298 (61.1) | 727 (62.0) |  |
| Age >75, n (%) | 463 (27.9) | 122 (25.0) | 341 (29.1) |  |
| NIHSS 0, n (%) | 177 (10.7) | 114 (23.5) | 63 (5.4) | < 0.0001 |
| NIHSS 1-4, n (%) | 1,116 (67.5) | 306 (63.0) | 810 (69.4) |  |
| NIHSS >4, n (%) | 360 (21.8) | 66 (13.6) | 294 (25.2) |  |
| mRS >2, n (%) | 608 (36.7) | 105 (21.5) | 503 (43) | < 0.0001 |
| Endovascular Thrombectomy, n (%) | 39 (2.4) | 9 (1.9) | 30 (2.6) | 0.2565 |
| Intravenous thrombolysis, n (%) | 361 (21.8) | 102 (20.9) | 259 (22.1) | 0.5958 |
| Heart Failure, n (%) | 48 (2.9) | 15 (3.1) | 33 (2.8) | 0.7729 |
| COPD, n (%) | 77 (4.7) | 16 (3.3) | 61 (5.2) | 0.0898 |
| Arterial Hypertension, n (%) | 1,281 (77.6) | 336 (69.4) | 945 (81.0) | < 0.0001 |
| Diabetes mellitus, n (%) | 440 (26.6) | 105 (21.6) | 335 (28.7) | 0.0030 |
| Hyperlipidemia, n (%) | 876 (53.1) | 230 (47.4) | 646 (55.4) | 0.0031 |
| Renal impairment, n (%) | 128 (7.8) | 30 (6.2) | 98 (8.4) | 0.1245 |
| Vascular disease, n (%) | 237 (14.4) | 59 (12.2) | 178 (15.3) | 0.1016 |
| Prior cardiovascular event, n (%) | 405 (24.6) | 107 (22.1) | 298 (25.6) | 0.1326 |
| Smoking, n (%) | 824 (49.9) | 222 (45.7) | 602 (51.6) | 0.0304 |
| BMI >30kg/m², n (%) | 412 (25.1) | 108 (22.4) | 304 (26.2) | 0.1037 |

**Supplementary Table 4.** Overview of further ECG abnormalities within the first 24 hours categorized by age groups. Provided are number (%) and median (25^th^; 75^th^ percentile) and p-value as calculated by chi square test.

| **ECG finding (24 hours)** | **Total**  **N=1,665** | **<50 years**  **N=175** | **50-75 years**  **N=1,026** | **>75 years**  **N=464** | **p-value** |
| --- | --- | --- | --- | --- | --- |
| **Premature atrial complexes** | 1,633 (98.1%) | 163 (93.1%) | 1,012 (98.6%) | 458 (98.7%) | 0.0003 |
| **Supraventricular ectopic couplets** | 1,135 (68.2%) | 72 (41.1%) | 679 (66.2%) | 384 (82.8) | < 0.0001 |
| **Supraventricular ectopic run** | 915 (55.0%) | 47 (26.9%) | 537 (52.3%) | 331 (71.3%) | < 0.0001 |
| **Supraventricular tachycardia (SVT)** | 68 (4.1%) | 4 (2.3%) | 39 (3.8%) | 25 (5.4%) | 0.1598 |
| **Atrial fibrillation (AF)** | 36 (2.2%) | 0 (0.0%) | 16 (1.6%) | 20 (4.3%) | 0.0004 |
| **Premature ventricular complex (PVC)** | 1,428 (85.8%) | 116 (66.3%) | 885 (86.3%) | 427 (92.0%) | < 0.0001 |
| **Ventricular ectopic couplet** | 466 (28.0%) | 20 (11.4%) | 275 (26.8%) | 171 (36.9%) | < 0.0001 |
| **Ventricular bigeminy** | 233 (14.0%) | 8 (4.6%) | 129 (12.6%) | 96 (20.7%) | < 0.0001 |
| **Ventricular trigeminy** | 241 (14.5%) | 10 (5.7%) | 135 (13.2%) | 96 (20.7%) | < 0.0001 |
| **R-on-T phenomenon** | 84 (5.0%) | 1 (0.6%) | 45 (4.4%) | 38 (8.2%) | 0.0001 |
| **Ventricular run/accelerated idioventricular rhythm** | 97 (5.8%) | 3 (1.7%) | 58 (5.7%) | 36 (7.8%) | 0.0135 |
| **Ventricular ectopic triplet** | 152 (9.1%) | 1 (0.6%) | 94 (9.2%) | 57 (12.3%) | < 0.0001 |
| **Ventricular tachycardia** | 28 (1.7%) | 2 (1.1%) | 18 (1.8%) | 8 (1.7%) | 0.8310 |
| **Tachycardia** | 245 (14.7%) | 55 (31.4%) | 128 (12.5%) | 62 (13.4) | < 0.0001 |
| **Bradycardia** | 336 (20.2%) | 46 (26.3%) | 206 (20.1%) | 84 (18.1%) | 0.0707 |
| **Pause** | 33 (2.0%) | 3 (1.7%) | 17 (1.7%) | 13 (2.8%) | 0.3283 |
| **Supraventricular escape** | 430 (25.8%) | 46 (26.3%) | 245 (23.9%) | 139 (30.0%) | 0.0454 |
| **Ventricular escape** | 118 (7.1%) | 6 (3.4%) | 56 (5.5%) | 56 (12.1%) | < 0.0001 |

**Supplementary Table 5.** Overview of further ECG abnormalities within the first 24 hours categorized by sex. Provided are absolute number (%).

| **ECG finding (24 hours)** | **Total**  **N=1,665** | **Male**  **N = 993 (59.6%)** | **Female**  **N = 672 (40.4%)** | **p-value** |
| --- | --- | --- | --- | --- |
| **Premature atrial complex** | 1,633 (98.1%) | 977 (98.4%) | 656 (97.6%) | 0.2617 |
| **Supraventricular ectopic couplets** | 1,135 (68.2%) | 653 (65.8%) | 482 (71.7%) | 0.0103 |
| **Supraventricular ectopic run** | 915 (55.0%) | 520 (52.4%) | 395 (58.8%) | 0.0099 |
| **Supraventricular tachycardia (SVT)** | 68 (4.1%) | 34 (3.4%) | 34 (5.1%) | 0.0981 |
| **Atrial fibrillation (AF)** | 36 (2.2%) | 17 (1.7%) | 19 (2.8%) | 0.1247 |
| **Premature ventricular complex (PVC)** | 1,428 (85.8%) | 868 (87.4%) | 560 (83.3%) | 0.0194 |
| **Ventricular ectopic couplet** | 466 (28.0%) | 312 (31.4%) | 154 (22.9%) | 0.0001 |
| **Ventricular bigeminy** | 233 (14.0%) | 158 (15.9%) | 75 (11.2%) | 0.0061 |
| **Ventricular trigeminy** | 241 (14.5%) | 164 (16.5%) | 77 (11.5%) | 0.0040 |
| **R-on-T phenomenon** | 84 (5.0%) | 56 (5.6%) | 28 (4.2%) | 0.4211 |
| **Ventricular run/accelerated idioventricular rhythm (AIVR)** | 97 (5.8%) | 64 (6.4%) | 33 (4.9%) | 0.1897 |
| **Ventricular ectopic triplet** | 152 (9.1%) | 97 (9.8%) | 55 (8.2%) | 0.2709 |
| **Ventricular tachycardia (VT)** | 28 (1.7%) | 22 (2.2%) | 6 (0.9%) | 0.0395 |
| **Tachycardia** | 245 (14.7%) | 119 (12.0%) | 126 (18.8%) | 0.0001 |
| **Bradycardia** | 336 (20.2%) | 221 (22.3%) | 115 (17.1%) | 0.0103 |
| **Pause** | 33 (2.0%) | 25 (2.5%) | 8 (1.2%) | 0.0566 |
| **Supraventricular escape (SVEsc)** | 430 (25.8%) | 267 (26.9%) | 163 (24.3%) | 0.2286 |
| **Ventricular escape (VEsc)** | 118 (7.1%) | 72 (7.3%) | 46 (6.8%) | 0.7517 |

**Supplementary Table 6.** Overview of further ECG abnormalities within the first 24 hours divided in stroke and TIA cases. Provided are absolute number (%).

| **ECG finding (24 hours)** | **Total**  **N=1,661 (4 missing)** | **TIA**  **N = 488 (29.4%)** | **Stroke**  **N = 1,173 (70.6%)** | **p-value** |
| --- | --- | --- | --- | --- |
| **Premature atrial complex** | 1,629 (98.1%) | 477 (97.7%) | 1,152 (98.2%) | 0.5310 |
| **Supraventricular ectopic couplets** | 1,132 (68.2%) | 331 (67.8%) | 801 (68.2%) | 0.8550 |
| **Supraventricular ectopic run** | 914 (55.0%) | 254 (52.0%) | 660 (56.3%) | 0.1156 |
| **Supraventricular tachycardia (SVT)** | 68 (4.1%) | 24 (4.9%) | 44 (3.8%) | 0.2743 |
| **Atrial fibrillation (AF)** | 36 (2.2%) | 10 (2.0%) | 26 (2.2%) | 0.8310 |
| **Premature ventricular complex (PVC)** | 1,426 (85.9%) | 419 (85.9%) | 1,007 (85.8%) | 0.9947 |
| **Ventricular ectopic couplet** | 465 (28.0%) | 124 (25.4%) | 341 (29.1%) | 0.1301 |
| **Ventricular bigeminy** | 233 (14.0%) | 71 (14.5%) | 162 (13.8%) | 0.6930 |
| **Ventricular trigeminy** | 241 (14.5%) | 62 (12.7%) | 179 (15.3%) | 0.1780 |
| **R-on-T phenomenon** | 84 (5.1%) | 23 (4.7%) | 61 (5.2%) | 0.6798 |
| **Ventricular run/accelerated idioventricular rhythm (AIVR)** | 97 (5.8%) | 22 (4.5%) | 75 (6.4%) | 0.1355 |
| **Ventricular ectopic triplet** | 152 (9.2%) | 40 (8.2%) | 112 (9.5%) | 0.3842 |
| **Ventricular tachycardia (VT)** | 28 (1.7%) | 7 (1.4%) | 21 (1.8%) | 0.6078 |
| **Tachycardia** | 244 (14.7%) | 74 (15.2%) | 170 (14.5%) | 0.7249 |
| **Bradycardia** | 335 (20.2%) | 129 (26.4%) | 206 (17.6%) | < 0.0001 |
| **Pause** | 33 (2.0%) | 7 (1.4%) | 26 (2.2%) | 0.2981 |
| **Supraventricular escape (SVEsc)** | 429 (25.8%) | 140 (28.7%) | 289 (24.6%) | 0.0858 |
| **Ventricular escape (VEsc)** | 118 (7.1%) | 40 (8.2%) | 78 (6.6%) | 0.2636 |

**Supplementary Table 7.** Overview of ECG findings investigated and their clinical relevance based on the literature.

| **ECG finding** | **Clinical Relevance** |
| --- | --- |
| **Supraventricular ectopic beats (SVE)** | Ubiquitous even in absence of cardiac disease^29^; higher frequency associated with AF, stroke and mortality^12,30-32^ |
| **Supraventricular ectopic couplet** | See Supraventricular ectopic beats (SVE) |
| **Supraventricular ectopic run** | See Supraventricular ectopic beats (SVE) |
| **Premature ventricular complex (PVC)** | High prevalence in general population; association with cardiovascular disease (e.g. coronary artery disease and myocardial infarction) and mortality^33-36^ |
| **Ventricular ectopic couplet** | See Premature ventricular complex (PVC) |
| **Ventricular ectopic triplet** | See Premature ventricular complex (PVC) |
| **Ventricular tachycardia (VT)** | Potentially life-threatening arrhythmia when sustained; incidental detection of non-sustained VT in the absence of underlying cardiac disease of unknown relevance^35,37-39^ |
| **Tachycardia** | Spectrum of causes with varying clinical significance; association of elevated resting heart rate with mortality ^15,40,41^ |
| **Bradycardia** | Spectrum of causes with varying clinical significance; sinus bradycardia usually without adverse prognostic significance^42-44^ |
| **Pause** | Spectrum of causes with varying clinical significance (e.g. sinus pause, atrioventricular block)^43,44^ |
| **Supraventricular escape** | Indicator of atrial, especially sinus node dysfunction^45^ |
| **Ventricular escape (VEsc)** | Indicator of atrial or AV node dysfunction |
| **R-on-T phenomenon** | PVC initiation in the “vulnerable period” potentially leading to malignant ventricular arrhythmias^46^ |
| **Supraventricular tachycardia (SVT)** | Spectrum of causes with varying clinical significance (e.g. atrial and AV nodal disorders) |
| **Ventricular run/accelerated idioventricular rhythm** | See Premature ventricular complex (PVC) |
| **Ventricular bigeminy** | See Premature ventricular complex (PVC) |
| **Ventricular trigeminy** | See Premature ventricular complex (PVC) |
| **Atrial fibrillation (AF)** | Association with increased morbidity and mortality, e.g. through increased risk of stroke and heart failure^5^ |

**Supplementary Table 8.** Overview ECG findings with respective definition used for core laboratory classification

| **Abnormal ECG finding** | **Definition** |
| --- | --- |
| **Premature atrial complex** | isolated premature ectopic supraventricular beats |
| **Supraventricular ectopic couplets** | two consecutive supraventricular ectopic beats |
| **Supraventricular ectopic run** | three or more consecutive supraventricular ectopic beats |
| **Supraventricular tachycardia** | >180 bpm, <30 s |
| **Atrial fibrillation** | Atrial fibrillation ≥30 s |
| **Premature ventricular complex** | isolated ventricular ectopic beats |
| **Ventricular ectopic couplet** | two consecutive ventricular ectopic beats |
| **Ventricular bigeminy** | repetitive patterns in which every second beat was ventricular |
| **Ventricular trigeminy** | repetitive patterns in which every third beat was ventricular |
| **R-on-T phenomenon** | premature ventricular complex superimposed on the T wave of the preceding beat |
| **Ventricular run/accelerated idioventricular rhythm (AIVR)** | ≥3 consecutive ventricular beats usually with gradual onset |
| **Ventricular ectopic triplet** | three consecutive supraventricular ectopic beats |
| **Non-sustained ventricular tachycardia** | 4–30 beats |
| **Sustained ventricular tachycardia** | >30 beats |
| **Supraventricular escape (SVEsc)** | delayed subsidiary supraventricular beat occurring after a pause |
| **Ventricular escape (VEsc)** | delayed subsidiary ventricular beat occurring after a pause |
| **Pause** | ≥5 s |
| **Bradycardia** | <60 bpm |
| **Tachycardia** | ≥100 bpm |
